# Cell-Type-Specific Neuroproteomics of Synapses

**DOI:** 10.3390/biom13060998

**Published:** 2023-06-16

**Authors:** Yun Young Yim, Eric J. Nestler

**Affiliations:** Nash Family Department of Neuroscience and Friedman Brain Institute, Icahn School of Medicine at Mount Sinai, New York, NY 10029, USA; eric.nestler@mssm.edu

**Keywords:** neuroproteomics, synapse, neurological and psychiatric disorders, cell-type specificity

## Abstract

In the last two decades, our knowledge of synaptic proteomes and their relationship to normal brain function and neuropsychiatric disorders has been expanding rapidly through the use of more powerful neuroproteomic approaches. However, mass spectrometry (MS)-based neuroproteomic studies of synapses still require cell-type, spatial, and temporal proteome information. With the advancement of sample preparation and MS techniques, we have just begun to identify and understand proteomes within a given cell type, subcellular compartment, and cell-type-specific synapse. Here, we review the progress and limitations of MS-based neuroproteomics of synapses in the mammalian CNS and highlight the recent applications of these approaches in studying neuropsychiatric disorders such as major depressive disorder and substance use disorders. Combining neuroproteomic findings with other omics studies can generate an in-depth, comprehensive map of synaptic proteomes and possibly identify new therapeutic targets and biomarkers for several central nervous system disorders.

## 1. Introduction

The application of proteomic analyses in neuroscience has significantly increased in the past two decades [1,2]. Historically, genomic and transcriptomic analyses were extensively used to search for mutations in patients’ genomes or changes in gene expression in neuropsychiatric disorders such as autism spectrum disorder, Alzheimer’s disease, and schizophrenia [3]. However, due to the molecular complexity and heterogeneity of each of these disorders and the lack of strong coincidence between mRNA and protein expression levels, genetic and transcriptomic findings fail to fully explain the pathophysiological mechanisms of these syndromes. This discrepancy raises the need for an alternative omics approach, such as proteomics, to directly examine levels of individual proteins under these conditions.

Proteomics is the study of the proteome, the comprehensive set of proteins expressed by a genome in a cell, and neuroproteomics is the study of proteomes in the nervous system [4]. Unlike the proteomic analysis of other tissues, neuroproteomics is particularly challenging due to the need for cell-type-, region-, and temporal-specific analyses. To identify proteins in the central nervous system (CNS) and peripheral nervous system (PNS), understand their interactions, identify post-translational modifications, and discover potential biomarkers, neuroproteomic investigations require the conjunction of many biochemical techniques, including sample separation, gel electrophoresis, liquid chromatography, and mass spectrometry, and bioinformatics analyses. Several excellent reviews cover the applications and limitations of several neuroproteomic techniques [1,5,6,7,8] and synaptic neuroproteomics of PNS [9,10,11,12] and other model organisms [13,14]. This review will focus on mass spectrometry (MS)-based neuroproteomics of synapses in mammalian CNS.

Synapses interconnect approximately 86 billion neurons in a human brain, forming neural circuits [15], and mediate neuronal communication, resulting behavioral function. There are two different types of synapses, electrical and chemical, but the large majority of mammalian synapses are chemical and use neurotransmitters and neuropeptides [15]. A chemical synapse generally comprises three main constituents, a presynaptic terminal, a synaptic cleft, and a postsynaptic compartment. It contains approximately 1000–1500 distinct proteins with a turnover rate of 0.7% per hour [16].

Two decades of MS-based synaptic neuroproteomic studies have identified over a thousand synaptic proteins, tens of thousands of phosphorylation sites, and provided transient and time-resolved information on protein–protein interactions and structures. In 2019, synapse-specific gene ontology (SynGo) classification was established using published, expert-curated annotations. SynGo contains 87 synaptic locations and 179 synaptic processes and showed that genes that encode synaptic proteins are exceptionally well conserved and less tolerant to mutations than other genes [17]. In 2021, the synaptic proteome SQLite database, integrating 58 published synaptic proteomic datasets, was also established. It covered 8087 unique genes and 407,643 direct protein interactions [18]. Overall, MS-based synaptic neuroproteomic studies have significantly expanded our understanding of synapses not only in normal brain function but also in the pathophysiology of CNS disorders [19], especially based on the unbiased nature of these approaches [20,21,22,23]. However, there are several limitations to these studies.

We still lack cell-type-, subcellular compartment-, and synapse-cell-type-specific proteome information, with neuroproteomic studies of cell types, subcellular compartments, and cell-type-specific synapses now only just beginning [23,24,25,26,27]. Here, we focus on advances in MS-based neuroproteomic studies of chemical synapses. We highlight the recent application of these methods to specific cell types and subcellular compartments and cell-type-specific synapses in the context of CNS disorders. Integrating neuroproteomic approaches with other omics will improve our understanding of synapses and ultimately lead to the identification of biomarkers or new therapeutic targets.

## 2. Synapses

At chemical synapses, depolarizing electrical signals are rapidly converted into chemical signals by opening voltage-dependent Ca^2+^ channels, which allow calcium influx and change the local Ca^2+^ concentration critical for the release of synaptic vesicles (SVs) and large dense-core vesicles (LDCVs) [28,29,30]. The fusion of the vesicle membrane with the plasma membrane, leading to the release of the soluble contents of synaptic vesicles (SVs) and large dense-core vesicles (LDCVs), is initiated by the interaction between soluble NSF attachment protein receptor (SNARE) proteins present on the plasma membrane (t-SNARE) and vesicle (v-SNARE) [31,32,33,34,35]. This process is facilitated by the increased levels of Ca^2+^ in the presynaptic terminal [31,32,33,34,35]. Once released, neurotransmitters bind and activate their receptors located predominately in the postsynaptic membrane. The majority of the receptors are ligand-gated ion channels, with minor populations of G protein-coupled receptors in a small number of synapses. [29,36,37]. The intricate mechanisms underlying synaptic transmission and the release of large dense-core vesicles (LDCVs) have been comprehensively explored and elucidated in numerous outstanding reviews [29,34,35,37,38,39]. Here, we focus on the structure and isolation of synapses.

### 2.1. Structure of Synapses

Synapses are structurally complex despite being small. Classically, synapses were described as bipartite, containing pre- and postsynaptic compartments [15]. Now, with advancements in understanding of the bidirectional communication between neurons and astrocytes and the role of the extracellular matrix (ECM) in regulating synaptic functions, tripartite [40,41] and tetrapartite [42,43,44] synapses, in addition to bipartite synapses, have been widely studied. A tripartite synapse is one with pre- and postsynaptic neuronal compartments plus astrocytes, while a tetrapartite synapse includes the ECM as well. At these synapses, astrocytes and the ECM regulate both structural and functional aspects of synaptic plasticity. An in-depth discussion of tripartite and tetrapartite synapses is beyond the scope of this review, in which we focus mainly on bipartite synapses.

**Figure 1:**
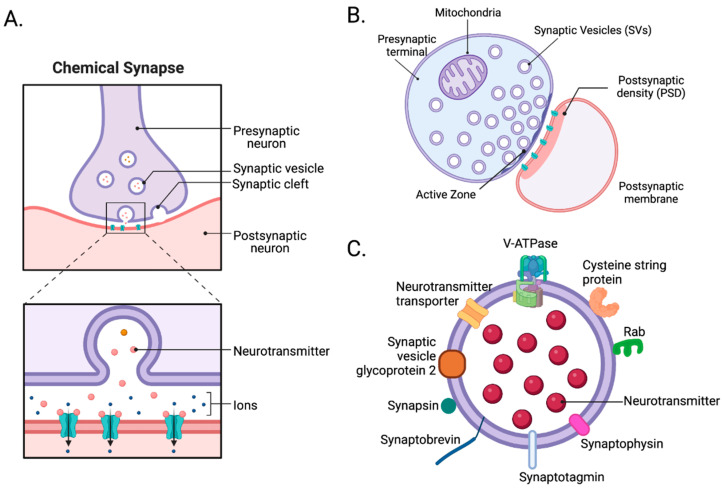
The architecture of synapses. (**A**) At chemical synapses, depolarizing electrical signals are rapidly converted into chemical signals by neurotransmitters released through exocytosis from the presynaptic terminal. Once released, they bind and activate receptors and channels on the postsynaptic membrane to initiate excitatory or inhibitory postsynaptic currents in postsynaptic cells. (**B**) A synaptosome is a “pinched-off nerve ending” consisting of a presynaptic terminal with its active zone, synaptic cleft, and postsynaptic membrane with its postsynaptic density (PSD). (**C**) Synaptic vesicles, the carriers of neurotransmitters, contain numerous proteins, such as V-ATPase, neurotransmitter transporter, cysteine string protein (CSP), RAB, synaptophysin, synaptotagmin, and synaptobrevin [56]. Created with BioRender.com (Shiz Aoki, Toronto, ON, Canada).

As noted, bipartite synapses contain three components, a presynaptic nerve terminal, a postsynaptic compartment, and a synaptic cleft. The presynaptic terminal includes its plasma membrane, which contains an active zone (Figure 1A) where vesicle mobilization, docking, priming, exocytosis, and endocytosis occur [45]. Both excitatory and inhibitory presynaptic terminals share similar structures. Differences primarily lie in neurotransmitter-synthesizing enzymes and transporters. Within the presynaptic terminal, numerous synaptic proteins, such as the SNARE complex and synaptotagmins, mediate the fusion of vesicles with the plasma membrane and are expressed in both excitatory and inhibitory synapses [46]. The postsynaptic compartment includes the postsynaptic plasma membrane containing the postsynaptic density (PSD). PSDs are where cell surface proteins, neurotransmitter receptors, cell-adhesion molecules, intracellular signaling molecules, and cytoskeletal filaments are densely present [47]. Unlike presynaptic terminals, postsynaptic compartments of excitatory and inhibitory synapses are more intrinsically different. In excitatory postsynaptic compartments, which typically represent the heads of dendritic spines, PSD95, SHANK, HOMER, inotropic glutamate receptors (AMPA, NMDA, and kainite-type receptors), and calcium–calmodulin-dependent protein kinase 2 (CaMK2), among many other proteins, are present [47]. By contrast, in inhibitory postsynaptic compartments, which typically occur on dendritic shafts, gephyrin, collybistin, and ionotropic GABA receptors, among many other proteins, are expressed [48]. Lastly, the synaptic cleft—the space between the pre- and postsynaptic compartments—is a protein-rich environment whose components can drive synaptogenesis and modulate synaptic maturation and transmission [39]. However, biochemical isolation of the synaptic cleft is very complicated. With advances in electron microscopy, proteomics, biotin labeling, and other biochemical approaches, researchers identified numerous proteins, including synapse-organizing adhesion proteins, such as ephrin, cadherin, and neurexins, which reside in the synaptic cleft. They also uncovered differences between excitatory glutamatergic and inhibitory GABAergic synaptic clefts [49,50]. For example, glutamatergic synaptic clefts contain NLGN1, LRRTM1, and LRRTM2 [51,52], while GABAergic synaptic clefts contain SLITRK3 and NLGN2 [53,54,55]. In glutamatergic synaptic clefts, postsynaptic NLGN1 interacts with presynaptic neurexins. However, in GABAergic synaptic clefts, postsynaptic NLGN2 interacts with presynaptic neurexins [49].

### 2.2. Isolation of Synapses

Biochemical isolation of synapses or SVs, followed by neuroproteomic analysis, is commonly utilized to understand the architecture of synapses and the molecular mechanisms of synaptic transmission in the brain [57].

Synaptosomes are “pinched-off nerve endings” composed of several cellular fragments, including a presynaptic nerve terminal with its active zone, mitochondria, SVs, plus the associated postsynaptic membrane with its PSD (Figure 1B) [46]. To prepare synaptosomes, brain tissue is first homogenized in an isotonic sucrose solution. The homogenate is centrifuged at various speeds to remove nuclei, cytosol, and cellular debris. Then, depending on the type of experiment, crude synaptosomes are further purified using discontinuous sucrose [58,59], ficoll [60,61], or percoll [62] gradient ultracentrifugation to remove mitochondria and myelin. Additionally, crude synaptosomes can be further fractionated to obtain synaptic sub-compartments, such as presynaptic nerve terminals, postsynaptic membranes, PSDs, and synaptic cytosol. Synaptosomes are the perfect model for understanding synaptic transmission and cataloging synaptic proteins. However, the applications of synaptosomes may require careful consideration of the following limitations. The average synaptosome diameter ranges from 0.5 to 0.9 µm [57], which makes fluorescence imaging and sorting challenging. In addition, commonly used molecular techniques, such as transfections of tagged genes or RNAi knockdown, cannot be applied to synaptosomes. Instead, synaptic protein manipulation must be conducted prior to brain tissue collection. Synaptosomes also need to be depolarized by chemicals since they are not sufficiently responsive to field stimulation [57]. Despite these limitations, synaptosomes are widely used, especially in neuroproteomic studies. In Wilhelm et al., 60 proteins were localized in their respective copy numbers within a three-dimensional model of an average synapse. In total, approximately 300,000 protein molecules were found in an average synapse, including multiple copies of numerous transporters, receptors, and ion channels, along with vesicle trafficking proteins (e.g., SNAP25, VAMP2, and syntaxin1) and other presynaptic proteins critical for exocytosis (e.g., SEC1/MUNC18 [SM] proteins and MUNC13 [63]).

SVs are essential organelles in the presynaptic terminal for neurotransmission, and SV integral or membrane-associated proteins mediate the various functions. These functions include organelle transport, interactions with the nerve terminal cytoskeleton, and regulated interactions with the presynaptic plasma membrane during exo- and endocytosis, which affect synaptic function and pathophysiology (Figure 1C) [25,64]. Understanding the composition of SVs and their trafficking mechanisms is essential to understanding synaptic transmission. In neuroproteomic and other biochemical studies, three different SV isolation protocols are widely used. One involves subcellular fractionation of crude synaptosomes, and the other involves direct isolation of SVs from brain homogenates using differential and density-gradient centrifugation [65,66]. Since the 1960s, centrifugation methods used to isolate SVs have evolved to improve the yield and purity of SVs. However, today’s centrifugation methods still suffer from low final yields, low purity, and longer preparation time. Due to the small size of SVs, approximately 40–50 nm in diameter, their centrifugation purification takes ~24 h. Recently, immunoprecipitation (IP) has been more favorable in isolating SVs. Using an SV tag, such as RHO1D4, synaptotagmin1, or SV glycoprotein 2A/B/C [25,67], the IP method only takes ~2 h and is more selective than centrifugation [25]. With these advancements, several studies have successfully identified SV proteomes.

With the advent of single-cell and cell-type-specific transcriptomic techniques, neuroproteomics is now shifting toward identifying proteome changes with spatial and temporal information. While focusing on a specific brain region provides some spatial resolution, this fails to provide the cell-type- and synapse-cell-type-specific and temporal information. For this reason, we focus here on the efforts to accomplish the latter.

## 3. Advancements in Neuroproteomics

Most neuroproteomic studies to date have used entire brain region or large sections of the brain and, therefore, yield averaged proteome changes. Given the importance of more precise analyses for understanding a role of specific protein in the CNS, the field is pursuing numerous technical innovations, as described below.

### 3.1. Isolation of Cell Types, Subcellular Compartments, and Cell-Type-Specific Synapses

#### 3.1.1. Transgenic Animals

**Figure 2:**
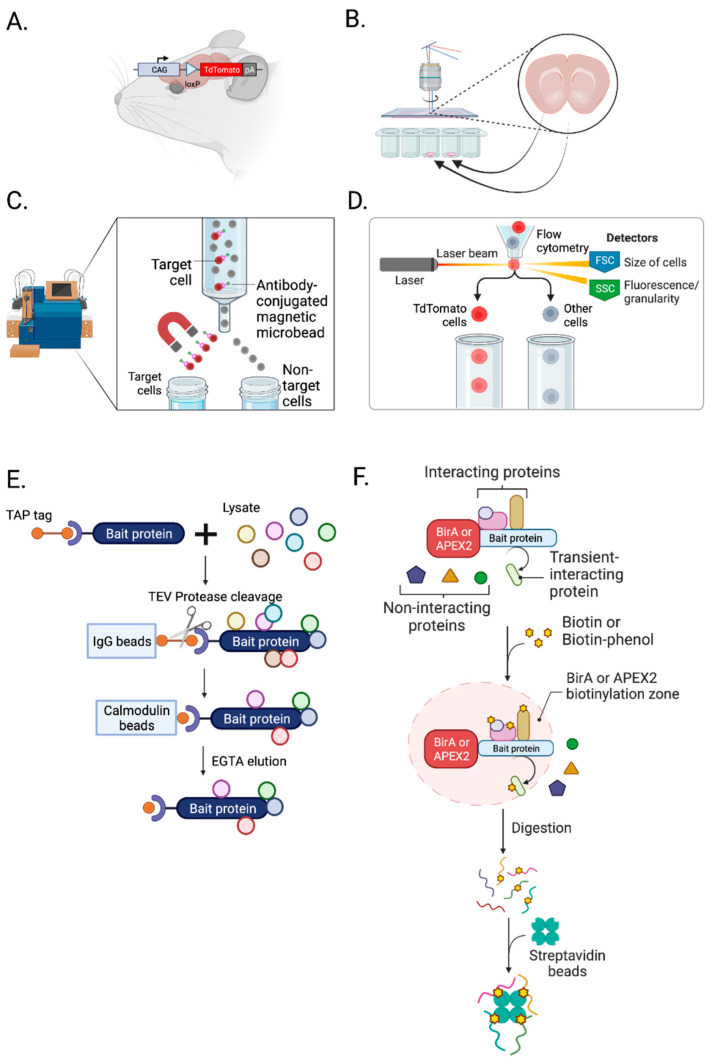
Isolation and enrichment of chemical synapses. (**A**) Crossbreeding a *loxP* “floxed” rodent with Cre driver rodent labels cell-type-specific synapses with fluorophores, such as TdTomato. (**B**) Laser capture microdissection (LCM) can isolate a subpopulation of cells from a brain slice. (**C**) Magnetic-activated cell sorting (MACS) is a bulk isolation technique utilizing magnetic and cell surface markers. (**D**) Fluorescence-activated cell sorting (FACS) isolates cells and synaptosomes using a surface marker, size, and granularity. (**E**) Tandem affinity purification (TAP) uses TEV protease, and IgG and calmodulin beads to isolate not only a bait but also proteins interacting with a bait. (**F**) BioID and APEX2 proximity labeling techniques can label proteins in an activity-dependent manner. This labeling helps to identify neuroproteome changes and their interactions in a cell-type-, subcellular-compartment-, and activity-dependent manner. Created with BioRender.com (Shiz Aoki, Toronto, ON, Canada).

By crossbreeding fluorophore *loxP* “floxed” mice [68,69,70] or rats [70,71,72,73] with neuronal cell-type-specific Cre-expressing mice [74,75,76] or rats [77,78,79], we can label cell-type-specific synapses for MS-based neuroproteomic analyses and other biochemical assays (Figure 2A). In addition, Cre-dependent viruses can be applied to a specific brain region of neuron-specific Cre animals to label a subcellular compartment of synapses [67]. A list of available fluorophore-expressing *loxP* “floxed” and Cre recombinase animals can be found on the Jackson Laboratory website (https://www.jax.org/research-and-faculty/resources/cre-repository, (accessed on 10 May 2023)) and the Rat Resource and Research Center in the US (https://www.rrrc.us/, (accessed on 10 May 2023)).

#### 3.1.2. Laser Capture Microdissection (LCM)

LCM allows the isolation of a subpopulation of cells from tissue slices under direct microscopic visualization (Figure 2B) [80]. With 20 µm spatial resolution, it can isolate cells and specific subcellular compartments [81]. For example, LCM can dissect neuromelanin granules from substantia nigra [82] and separate neurons and amyloid plaques from Alzheimer’s disease brain tissue [83]. LCM is well validated and commonly used in transcriptomic studies but is yet to be widely used in neuroproteomic analysis, with its application limited primarily to human postmortem brain tissue [84,85,86,87,88]. Recently, LCM was applied to rat hippocampus to examine spatial proteomic changes [89]. In their study, do Canto et al. identified new signaling pathways and proteins present in specific layers and regions of the dentate gyrus. With micro-proteomics, which requires only 5000 cells [90] or nanoPOTs (see single-cell mass spectrometry), LCM has great potential for future neuroproteomic studies.

#### 3.1.3. Magnetic-Activated Cell Sorting (MACS)

MACS, known as immunomagnetic cell separation, isolates specific cell types using tiny paramagnetic beads coupled to antibodies, enzymes, lectins, or streptavidin (Figure 2C) [91]. It is one of the most common, inexpensive, user-friendly cell separation techniques. It does not require specialized training like fluorescence-activated cell sorting (FACS) and eliminates the need for fluorophores [92]. However, it involves bulk isolation compared to FACS, which provides cell-by-cell isolation [93]. Since 1990, MACS has been widely used in neuroscience to purify several CNS cell types (neurons, astrocytes, oligodendrocytes, and microglia) from a rodent brain [26,92,94]. MACS is particularly favorable for astrocytes [95,96]. Holt et al. showed that astrocytes isolated by MACS are significantly more morphologically complex than those isolated by FACS, suggesting that MACS is gentler than FACS [92]. However, isolating neurons from an adult rodent brain using MACS results in significant contamination [92]. This major limitation of MACS has made FACS much more commonly used for neuronal neuroproteomic studies.

#### 3.1.4. Fluorescence-Activated Cell Sorting (FACS)

FACS is a technique to isolate a homogeneous population from a heterogeneous cell population (Figure 2D). Samples are placed in a fluid stream, enter the flow cell in a cell-by-cell form through a nozzle and pass by a set of lasers, and the light scattering and fluorescence signals of each particle passing by are detected [97]. Then, individual cell types are collected into homogeneous fractions. FACS can isolate cells based on their surface marker, size, and granularity, and it allows the enrichment of even low abundant subpopulations with high purity. Nevertheless, FACS has several limitations. It can only sort suspended cells and requires several hundreds of microliters to milliliters of samples [98]. In addition, it highly depends on fluorescence signal intensity, so fluorescence compensation is necessary to sort cells accurately. Despite these limitations, FACS is commonly used in neuroproteomics and preferred over MACS for studies of neurons and synaptosomes.

Because synaptosomes are heterogenous, an additional isolation step is necessary to isolate cell-type-specific synaptosomes. However, synaptosomes are much more challenging to sort than cells: they are an order of magnitude smaller than an average cell. To successfully conduct fluorescence-activated synaptosome sorting (FASS), size standards, a non-light-scattering-dependent detection method, and longer sorting times are required [99,100]. Biesemann et al. successfully isolated glutamatergic synaptosomes using FASS and identified 163 enriched proteins in sorted glutamatergic synaptosomes [100]. Moreover, Paget-Blanc et al. successfully characterized dopaminergic (DA) synapses, with 57 proteins specifically enriched, and revealed “DA hub synapses”—those adhered to glutamatergic, GABAergic, or cholinergic synapses [23]. FASS combined with highly sensitive MS is the most widely used approach to study cell-type-specific synapses.

#### 3.1.5. Tandem Affinity Purification

The specificity of affinity reagents, such as antibodies, peptides, and ligands, limits the isolation of synapses with affinity methods [101,102,103,104,105,106]. The recovery of the native complex is also low and potentially includes more contaminants. To overcome these weaknesses, a tandem affinity purification (TAP) tag was developed [107]. TAP is an IP-based purification technique (Figure 2E). Initially, it was made with two IgG binding domains of *Straphylococus aureus* protein A (ProtA), a tobacco etch virus (TEV) protease cleavage site, and calmodulin binding peptide (CBP) [107]. ProtA binds tightly to an IgG matrix, requiring TEV protease to elute material under native conditions. Then, elutants are incubated with calmodulin-coated beads in the presence of calcium, allowing the CBP of TAP to bind to the beads. After going through multiple washes, EGTA is used for elution. A protease cleavage site between two affinity tags allows rapid purification of complexes under native conditions. Despite its strength, the original TAP tag has some disadvantages. The calmodulin affinity step was inefficient since endogenous calmodulin in mammalian cells interferes with the binding of the target, causing poor protein recovery. In addition, the chelating agent used in elution can irreversibly interfere with the function of cation-dependent proteins. Lastly, the original TAP tag is relatively large, 21 kDa, and can potentially impair protein function [108]. Now, thirty alternative TAP tags with different combinations of affinity handles and lower kDa are available [108]. Both C- and N-terminus TAP tags are available to isolate the protein of interest (with its associated proteins) without impairing protein function [109]. Moreover, transgenic mice lines with TAP tags have been developed over a decade to study protein–protein interactions in disease models and signaling complexes of synapses. For example, TAP-tagged PSD95 knockin and PSD95 conditional TAP mice have been used to isolate postsynaptic compartments of synapses and perform proteomic analysis of PSD-95-associated complexes in the forebrain [109] and hippocampus and its CA3 subfield [110].

#### 3.1.6. Protein Labeling

To overcome the limitations of MACS and FACS, numerous protein-labeling techniques have been developed to conduct subcellular compartment and cell-type-specific proteomic analysis [111]. The two most commonly used techniques are metabolic labeling and proximity labeling.

Bio-orthogonal non-canonical amino acid tagging (BONCAT) is a metabolic label that enriches cell-specific proteomes. A mutant methionyl-tRNA synthetase (MetRS^L274G^) labels newly translated proteins with the non-canonical amino acid [112]. This tool is very powerful in labeling newly synthesized proteins in a cell-type-specific manner when applied with Cre-*loxP* transgenic animals. Following copper-catalyzed azide-alkyne ligation (CLICK chemistry), labeled proteins can be isolated and analyzed by MS. Alvarez-Castelao et al. developed a protocol that labels, purifies, and identifies cell-type-specific proteomes in a Cre-recombinase-inducible mouse line expressing a mutant L274GMetRS. The authors successfully detected 2384 distinct proteins in hippocampal excitatory neurons and 1687 distinct proteins in cerebellum inhibitory neurons [113,114].

An alternative protein labeling approach is proximity labeling [115]. Genetically-encoded labeling enzymes such as BioID [116], TurboID [117], APEX2 [118], and horse radish peroxidase (HRP) [49,119,120] can be expressed and localized to a specific subcellular compartment and modify a freely diffusing biotin (Figure 2F). In situ biotinylation occurs rapidly from minutes to hours for TurboID and within seconds for APEX2 [121]. Then, with streptavidin affinity purification, labeled proteins can be isolated.

Although these methods extensively examine both cell-type- and subcellular-specific proteomes, several limitations must be addressed. Proximity labeling requires the expression of an exogenous enzyme and uses a transfection method or knockin mouse line [49]. In addition, endogenous biotinylation must be considered [122]. Currently, the application of BioID [123,124] or TurboID [125,126] to the brain of transgenic mice is very limited. To map activity-dependent changes at the proteome level, APEX2, known as fast proximity labeling, combined with Cre transgenic animals, is more favorable [121,127,128]. Despite the toxicity of biotin-phenol, H_2_O_2_, and the ex vivo application of APEX2 and HRP-mediated biotinylation, APEX2 and HRP labeling helps to identify neuroproteome changes in a cell-type-, subcellular-compartment-, and activity-dependent manner. Hobson et al. examined the DA presynaptic proteome using synaptosomes purified from the striatum of DAT-IRES-CRE mice expressing APEX2NES. From striatal synaptosomes with APEX2 expression in midbrain DA neurons, they identified 1533 proteins, including those involved in DA synthesis, release, reuptake, and degradation. Moreover, Suster et al. showed an efficient ex vivo cell surface biotinylation in the brain using Cre-dependent expression of a membrane-targeted HRP. ARMH4 was identified as a critical cell surface protein required for Purkinje cell dendrite arborization in the cerebellum [120].

### 3.2. Advancements in MS Approaches

**Figure 3:**
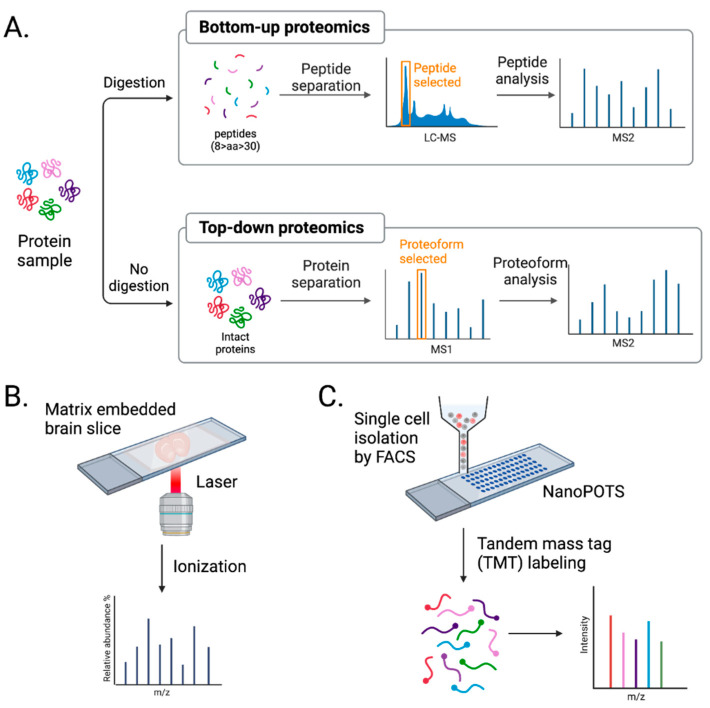
Advancement in mass spectrometry techniques. (**A**) MS-based proteomics is largely divided into bottom-up and top-down approaches. Bottom-up proteomics analyzes peptides, while top-down proteomics analyzes intact proteins. (**B**) Imaging mass spectrometry (IMS) provides a spatial distribution of molecules in a tissue sample. (**C**) Single-cell mass spectrometry (scMS) with NanoPOTs and FACS helps to unbiasedly study proteomes in single brain cells. Created with BioRender.com (Shiz Aoki, Toronto, ON, Canada).

The development of MS-based neuroproteomics has enabled the characterization and quantification of brain proteomes in a high throughput manner. MS-based proteomics is divided into two approaches: bottom-up vs. top-down (Figure 3A). The main difference is the digestion step.

Bottom-up proteomics uses proteases, such as trypsin, to digest proteins into peptides, which are then analyzed by MS (Figure 3A). The mass-to-charge ratio and predicted sequences of peptides are used to search a protein database to characterize the open-reading frame these isolated peptides are from. The pros of using bottom-up proteomics are that peptides are more easily separated by reverse-phase liquid chromatography, ionize well, and fragment in a more predictable manner [129]. However, the extensive use of proteases brings caveats. Peptides identified in bottom-up proteomics are often not specific to a single protein. Data must be filtered to identify unique peptides for a given protein to accurately identify and quantify that protein. In addition, the method only covers the partial sequence of a protein. Despite these limitations, bottom-up proteomics is the most commonly used MS approach, especially in neuroproteomics [112,130].

Top-down proteomics uses intact proteins and thereby eliminates issues caused by focusing on peptides. Intact proteins are fractionated and run on high-resolution MS, where proteoform, all of the different molecular forms in which the protein product of a single gene can be found, including genetic variations, alternatively spliced RNA transcripts, and post-translational modifications [131], are selected and analyzed (Figure 3A). This approach allows for 100% sequence coverage and complete characterization of proteomes, including genetic variation, alternative splicing, and post-translational modification [132]. However, several challenges, such as protein solubility, detection of low-abundance proteins, and proteome complexity, need to be addressed, and for these reasons, it is less favorable than bottom-up proteomics. With ongoing improvements in solubilizing membrane proteins and ECM, enriching low-abundance proteins, and separating intact proteins before MS analysis [133], the application of top-down proteomics in neuroproteomics will undoubtfully increase.

Since the mid-1990s, numerous proteomic methods have been developed and widely applied in a cell-type-specific manner in neuroscience [134]. Here, we highlight the two most trending methods of MS: imaging and single-cell MS.

#### 3.2.1. Direct In Situ Spatial Proteomics

Imaging mass spectrometry (IMS), such as matrix-assisted laser desorption/ionization IMS (MALDI-MS), provides a spatial distribution of molecules present in a sample (Figure 3B). MALDI-MS uses brain tissue embedded in a matrix, allowing the ionization of molecules in situ with a laser. Although suitable for de novo spatial proteome discovery, MALDI-MS suffers from shallow depth [2]. Proteins and peptides are challenging to ionize in this manner. Therefore, IMS is more applicable to studies of metabolites and lipids.

Imaging mass cytometry (IMC) with IMS is another approach that can be used to study cell types, subcellular compartments, and cell-type-specific synapses [2]. IMC uses antibodies coupled to heavy metal species to label proteins, allowing the simultaneous imaging of up to 40 different proteins, with labeled proteins identified by IMS [135,136]. In Van Deusen et al., protein-based cell atlases of the developing mouse telencephalon, diencephalon, mesencephalon, and rhombencephalon were mapped using this approach. They quantified 85 molecularly distinct cell populations, including neurons and myelin [136].

#### 3.2.2. Single-Cell Mass Spectrometry

Single-cell transcriptomics has transformed our understanding of the brain. However, mRNA and protein expression are often inconsistent [26,137,138,139]. This gap led to the development of single-cell mass spectrometry (scMS) [140]. Comparing the results of single-cell transcriptomics with single-cell proteomics can yield new insights into the mechanisms of circuit formation and function. With the advancement in liquid chromatography (LC), tandem mass spectrometry (MS/MS), and sample preparation, scMS has just begun to be applied in neuroproteomics.

Current MS-based proteomic approaches require samples containing a minimum of thousands of cells to provide in-depth profiling [90]. Because proteomics does not allow for amplification steps like PCR-based transcriptomics, many cells are required for proteomic analysis, which is the biggest hurdle of scMS. To overcome this, nanoPOTS, a nanodroplet process in one pot for trace samples, was developed [81]. nanoPOTS consists of two glass pieces, a slide and a spacer, which are micropatterned with hydrophilic nanowells surrounded by a hydrophobic surface. Nanowells serve as microreactors for cells or other protein samples. They can undergo chemical treatments to extract, reduce, alkylate, and digest in volumes as small as 200 nl while avoiding sample loss due to surface exposure. Using LCM and nanoPOTS, the quantitative profiling of >3000 proteins was achieved from 10 HeLa cells [81]. In addition, nearly 1000 proteins were detected from a 100 µm diameter section of a 12 µm thick slice of rat cerebral cortex [81].

scMS with multiplexed isobaric tandem mass tags (TMTs), including single-cell proteomics by mass spectrometry (ScoPE-MS) [141] and improved boosting to amplify the signal with isobaric labeling (iBASIL) strategy [142], are, so far, the most successful approaches used for single-cell, cell-type-specific proteomic analysis (Figure 3C). These methods enhance protein detection and minimize sample surface losses of labeled samples by using a pool of cells or standard peptides from proteins of interest, known as “carrier” or “boosting.” Both carrier and single cells are labeled with TMTs, adding the same total mass to the peptides but having a different isotope composition. This results in one isobaric mass signal on MS1 spectra, but once the peptide precursors are fragmented, the difference is found in the low *m/z* region in the MS2 or MS3 spectrum [143]. Then, the ratio of those reported ions from single cells quantifies the previously labeled sample. These methods allow the more accurate quantification of detected proteins compared to label-free proteomic analysis. However, quantifying proteins using these tags requires optimizing a carrier signal. Larger carrier proteomes may promote losses in quantifying low-abundance peptides. Furthermore, co-eluting and co-fragmented peptide signals may interfere with quantifying a peptide of interest. So far, these scMS efforts with TMTs have not been readily applied to study neural cells. Combining these with other cell-type-specific isolation techniques, such as FACS, will help characterize single brain cells [143]. Unbiased classification of neuron types by large-scale scMS and combining results with other available omics should improve quantification of brain proteomes.

## 4. Application of Neuroproteomic Analysis to Neuropsychiatric Disorders

Disordered functioning of synapses is known to contribute to a wide range of neuropsychiatric disorders [112]. Thus, an in-depth understanding of the molecular and functional organization of synapses and synaptic dysfunction in these neuropsychiatric disorders is essential.

Many high-throughput genomic and transcriptomic studies of such disorders have examined mutations in patient genomes or changes in their transcriptomes, yielding numerous key discoveries [144]. However, these approaches fail to offer a complete picture of disease states because they cannot detect the abundance of proteins and examine the protein networks. To fill this gap, neuroproteomics is increasingly used to discover biomarkers and explore underlying pathological mechanisms. Indeed, synaptic proteomic changes have been identified for several psychiatric disorders [145,146,147]. Here, we highlight studies across several disorders that use proteomic analysis of synapses to further refine the mechanisms of various disease states and identify new targets for possible treatments.

### 4.1. Autism Spectrum Disorder

Neurodevelopmental disorders are multifaceted conditions characterized by impairments in cognition, communication, behavior, and/or motor skills resulting from abnormal brain development [148]. Autism spectrum disorders (ASDs) are among the most well-studied neurodevelopmental disorders in neuroproteomics. ASDs are highly heritable, heterogeneous disorders characterized by impairments in social communication and sensory perception, often accompanied by repetitive behaviors [149]. Due to the varied genetic underpinnings of ASDs, the contribution of identified de novo mutations and rare or common variants found in ASDs is not always clear. Genetic variations associated with ASDs are highly enriched in genes encoding synaptic proteins, such as group 1 mGLURs, NMDARs, and SHANK, to name a few [149]. To further understand the signaling network of ASDs, various neuroproteomic approaches have been utilized [145,150,151,152,153,154,155,156].

SHANK3 is a large scaffold protein that organizes the PSD of glutamatergic synapses [155]. Mutation of *SHANK3* is hypothesized to perturb synaptic transmission in neural circuits throughout the brain and cause diverse neuropsychiatric phenotypes. With improvements in biochemical subcellular fractionation of synapses, the effect of *SHANK3* mutations was examined in striatal and hippocampal PSDs in *SHANK3* mutant mice [145]. Reim et al. identified changes in 55 and 61 proteins, out of ~2500, in striatal PSDs and hippocampal PSDs, respectively, from *SHANK3* mutants [145]. The findings of this study mirrored results from previous work using two different ASD mouse models, *Pten* mutant [157] and *Fmr1* knockout [150] mice. Together, the work highlights the value of unbiased and comprehensive screening of subcellular synapse anatomy in ASD-associated brain regions to understand the molecular consequences of the corresponding mutation and the big picture of ASD pathology [145]. Recently, using BioID and MS-based neuroproteomic approaches, protein–protein interaction (PPI) networks for 41 ASD risk genes were identified. The PPI network revealed the convergent pathways of ASDs as well as other pathways that are affected in only a subset of ASDs [156].

### 4.2. Alzheimer’s Disease

Alzheimer’s disease (AD), the most common form of progressive, age-related dementia, is a neurodegenerative disorder involving the gradual loss of synapses and the accumulation of amyloid β (Aβ) oligomers [158,159] and tau-containing neurofibrillary tangles [160,161]. Aβ and tau are also present in normal, healthy individuals, but under certain circumstances, which yet remain to be learned, Aβ and tau aggregate and start AD progression [162]. In AD patients, soluble Aβ and tau oligomers cause synaptic and cognitive dysfunctions by enhancing long-term depression (LTD) and inhibiting long-term potentiation (LTP), accelerating neuronal cell death [160,163,164,165,166]. Despite the well-known genetic underpinnings and molecular hallmarks of AD, treatment options for AD remain limited. Recent neuroproteomic analysis of AD synapses suggests new potential therapeutic targets.

Neuroproteomic analysis of synaptosomes from the human AD postmortem hippocampus and inferior temporal gyrus was first reported in 2013 [167]. Chang et al. identified expression changes in several synaptic proteins, such as synaptotagmin 1 and V-ATPase, located at the SV membrane. Kadoyama et al. later detected V-ATPase components in the hippocampus of bicuculline-treated App_osk_-Tg mice, a transgenic mouse model of AD. Moreover, the synaptic vulnerability caused by the genetic factor of sporadic AD, apolipoprotein E 4 alleles (*APOE4*), was identified using neuroproteomic analysis of superior temporal gyrus (BA41/42) and primary visual cortex (BA17) from human *APOE4+* brain tissue. In total, 5678 expressed proteins, including 1532 differentially expressed proteins important for synaptic and mitochondrial function, neuroimmune interactions, and intracellular signaling, were detected [168]. Cafeliello et al. is another exemplary study utilizing synaptosomes to identify local translation in TgCRND8 mice—another mouse model of AD. Using radioisotope labeling and BONCAT, this study showed that amyloid precursor protein (APP), which yields Aβ, is synaptically synthesized in the cerebral cortex and cerebellum of TgCRND8 mice. Overall, neuroproteomic analysis in AD studies has shed new light on AD pathophysiology and has suggested bicuculline, a GABA_A_ receptor blocker, as a potential treatment to improve cognition [169].

### 4.3. Schizophrenia

Schizophrenia (SCZ) is a heterogeneous psychotic disorder characterized by delusions, hallucinations, disorganized speech or behavior, and impaired cognitive ability [170]. The pathophysiology of SCZ is complex, and many factors are yet to be discovered [171]. SCZ involves numerous genetic loci and is highly pleiotropic [172]. Reduction in synaptic densities and abnormalities in neurotransmission are reported as pathophysiological signatures of SCZ [173,174,175,176,177]. Neuroproteomic analysis of the human SCZ postmortem brain revealed PSD proteins, such as SHANK3, MAPK3, and SNYPO, differentially expressed in SCZ [178]. Moreover, PPI and pathway analyses of proteomic experiments using primary hippocampal neurons treated with shRNA of SCZ risk genes, such as *TBR1*, *TCF4*, and *TOP3B*, suggested that syntaxin-mediated neurotransmitter release in SCZ may be affected owing to subtle dysregulation via an indirect upstream gene regulatory mechanism rather than dysregulation of the involved proteins, such as TCF4, TBR1, and TOP3B, per se [177]. These findings complement genomic analysis of schizophrenia risk genes that encode PSD proteins [154,179] and, most importantly, highlight the need for neuroproteomic studies to identify the network of protein changes.

### 4.4. Major Depressive Disorder

Major depressive disorder (MDD) is one of the most common mental disorders worldwide. In 2020, about 8.4% of all U.S. adults experienced at least one major depressive episode, and the lifetime prevalence is 17% [180]. It is also a multifactorial disorder. Studies suggest that MDD is caused by a combination of genetic predisposition (~35%) and environmental factors [181]. Our understanding of MDD‘s pathophysiology remains incomplete. Since up to 50% of MDD patients are not fully treated with available therapies, there is a tremendous unmet need for new therapeutics.

Proteome changes in MDD have been extensively studied. Postmortem anterior cingulate cortex [182], frontal cortex [183], and dorsolateral prefrontal cortex (DLPFC) [184] from MDD patients were analyzed with proteomic approaches. These studies have highlighted differentially expressed proteins involved in energy metabolisms, such as carbonic anhydrase, aldolase C, histidine triad nucleotide-binding proteins, and several subunits of oxidative phosphorylation complexes, in MDD. In addition, lower levels of adenosine triphosphatase (ATPase) were observed in MDD [184]. A phosphoproteomic study of DLPFC in MDD brains revealed differential phosphorylation levels of numerous synaptic proteins, including SNARE complex, SNAP25, and synapsin 1 [185]. Neuroproteomic analyses of cerebrospinal fluid [186] and plasma [187] from MDD patients have identified potential biomarkers. The discovery of biomarkers that can identify subtypes of MDD patients would be a major advance in the field.

### 4.5. Substance Use Disorders

The persistence of addiction is thought to be mediated by drug-induced changes in the physiology of reward-processing brain regions. Dysregulated signaling within brain reward regions, such as the nucleus accumbens (NAc), medial prefrontal cortex (mPFC), and basolateral amygdala (BLA), appears to play an especially critical role in promoting drug-seeking and relapse [27,188,189,190,191]. Determining these changes will reveal more effective targets for treating drug addiction and relapse. However, our understanding of the molecular mechanisms underlying these adaptations and alterations of signaling remains incomplete.

A broad-scale investigation of molecular alterations in brain reward regions through proteomics will help capture the biological basis of addiction-related behaviors. Recent neuroproteomics work has demonstrated that addiction-related behaviors emerge from converging subtle molecular changes. Bosch et al. showed 84 differentially regulated protein changes, including proteins with known roles in SVs and cytoskeleton in dorsal striatum synaptosomes of methamphetamine self-administering rats [192]. Furthermore, utilizing labeling techniques such as TMT and fluorophore, Lull et al. compared the PFC proteome in cocaine self-administering rats. They identified 20 significant changes, such as heat shock protein 73 and SNAP25 [193]. Recently, Puig et al. identified changes in 56 and 161 proteins from synaptosomes of postmortem NAc and DLPFC, respectively, in opioid use disorder patients. In NAc synaptosomes, proteins involved in inflammatory, mitochondria, and metabolic signaling pathways were identified. In contrast, proteins involved in inflammatory signaling, serotonergic, DA, cholinergic, and oxytocin neurotransmission were identified in DLPFC synaptosomes [194]. In both brain regions, proteins involved in GABAergic and glutamatergic synaptic functions, as well as circadian rhythms, were demonstrated, suggesting molecular disruption of circadian regulation of synaptic signaling in the human brain as a critical factor in opioid addiction [194]. Although these neuroproteomic studies have successfully identified critical intracellular signaling [195] and circuit-level networks [196] in synapses associated with drug-seeking, we still need to gain an understanding of neuronal cell-type-specific synaptic changes.

## 5. Limitations and Future Perspectives

With improvements in sample preparation and MS, MS-based proteomics has become an even more powerful tool in recent years. Throughout this review, we have highlighted the advantages and limitations of techniques used in MS neuroproteomics studies of synapses. In this section, we will highlight several limitations which must be considered, especially when examining the proteomic landscape of synapses in a cell-type-specific manner.

Unlike genomic technologies, which can capture the vast majority of all expressed RNAs, our ability to detect proteins remains limited. Out of perhaps tens of thousands of distinct types of proteins expressed in a given tissue, the best proteomic method can detect several thousand, with low-abundance proteins especially difficult to detect. This lack of sensitivity with proteomics is due to the inability to amplify signals as routinely performed for RNAs. Moreover, because only a partial protein sequence is used in most proteomic studies, proteins with low abundance, alternative splicing, alternative translation initiation sites, and point mutations are much more challenging to detect. In addition, a relatively large quantity of samples is needed in neuroproteomic studies. This is the biggest hurdle for neuroproteomics in investigating cell-type-specific synapses within a specific brain region. The top-down MS approach, in which intact proteins instead of peptides are analyzed, helps to overcome some of these issues regarding the partial sequence coverage. However, in top-down MS, it is difficult to accurately determine the monoisotopic mass and identify proteins larger than 50 kDa [112]. Further advancement in the sensitivity and resolution of MS technology [140,197,198,199], including the recent development of ultra-high sensitive true single-cell-derived proteomics (T-SCP) [200], along with associated enrichment and purification techniques, may close the gap between proteomics and other omics analyses.

Conducting cell-type-specific neuroproteomics is essential to advance the field. For example, in the NAc, two populations of medium spiny neurons (MSNs) generally exert opposite effects on behavior. D1-MSNs promote positive reinforcement and increase the formation of cocaine reward–context associations, whereas D2-MSNs appear to produce aversion and decrease cocaine reward [201,202]. Likewise, acute cocaine administration enhances D1-MSNs and suppresses D2-MSNs activity [203]. These cell types work in a subregion-dependent, complex, interweaving manner to drive drug-seeking and relapse behavior in the NAc [204,205,206]. To further understand substance-induced synaptic proteome changes in the brain’s reward circuitry, it is necessary to examine cocaine-induced D1- and D2-MSNs-specific synaptic proteome changes in the NAc. Such proteomic adaptations will likely drive reciprocal interactions between drug-induced transcriptional responses and synaptic dysfunction, perpetuating the “addiction cycle.” Delineating these complex reciprocal interactions will reveal more effective targets for treating drug addiction and relapse. Such advances will require technological improvements since current methods would require isolation of D1- or D2-MSNs from transgenic mice, where D1- or D2-MSNs are labeled with a fluorophore, for deep neuroproteomic analysis. Our lab has generated D1- or D2-MSNs labeled transgenic animals and isolated not only bipartite synapses of D1- or D2-MSNs but also tripartite synapses using FASS. By completing this study, we aim to demonstrate cell-type-specific synaptic changes in both bipartite and tripartite synapses.

With advances in imaging and genetic labeling methods, the spatiotemporal organization of synaptic proteins can now be visualized by identifying synaptic proteins at single-synapse resolution across mouse brain regions [207]. Proteomic characterization of PSD95-positive synapses have been conducted for 20 different human brain regions [208] and for mouse brain from postnatal day 1 to 18 months [209]. These studies help to understand the diversity of synapses as brain regions become dissimilar and shed light on how the protein constituents and architecture of synapses change through development. These studies also connect transcriptomic and neuroproteomic analyses to the structural synaptic development and plasticity of synapses within the subcellular compartment.

By integrating transcriptomics, translatomics, neuroproteomics, and super-resolution structural imaging, we are now at the next step of investigating the mechanistic links between behavioral changes, psychological function, and synaptic pathology associated with specific gene mutations in a particular brain region, with cell-type specificity and temporal information. Deep learning methods offer exciting promise for linking multi-omics and spatial data across cell types and structural organization [210]. An in-depth, comprehensive understanding of synaptic proteomes, especially in a cell-type-specific manner, along with the links between mRNA and protein, local regulation of protein synthesis, and changes in subsynaptic molecular organization, will expand the potential therapeutic targets for synapse-linked diseases. This understanding will not only correct abnormal neurotransmitter-mediated signaling but also change the translational perspectives of synaptic proteins.

## Data Availability

Not applicable.
